# Parking Lot Traffic Prediction Based on Fusion of Multifaceted Spatio-Temporal Features

**DOI:** 10.3390/s24154971

**Published:** 2024-07-31

**Authors:** Lechuan Zhang, Bin Wang, Qian Zhang, Sulei Zhu, Yan Ma

**Affiliations:** College of Information, Mechanical and Electrical Engineering, Shanghai Normal University, Shanghai 201400, China

**Keywords:** PGI, traffic flow, parking efficiency, multifaceted spatio-temporal features, spatio-temporal correlation, spatial-temporal graph

## Abstract

With the rapid growth of population and vehicles, issues such as traffic congestion are becoming increasingly apparent. Parking guidance and information (PGI) systems are becoming more critical, with one of the most important tasks being the prediction of traffic flow in parking lots. Predicting parking traffic can effectively improve parking efficiency and alleviate traffic congestion, traffic accidents, and other problems. However, due to the complex characteristics of parking spatio-temporal data, high levels of noise, and the intricate influence of external factors, there are three challenges to predicting parking traffic in a city effectively: (1) how to better model the nonlinear, asymmetric, and complex spatial relationships among parking lots; (2) how to model the temporal autocorrelation of parking flow more accurately for each parking lot, whether periodic or aperiodic; and (3) how to model the correlation between external influences, such as holiday weekends, POIs (points of interest), and weather factors. In this context, this paper proposes a parking lot traffic prediction model based on the fusion of multifaceted spatio-temporal features (MFF-STGCN). The model consists of a feature embedding module, a spatio-temporal attention mechanism module, and a spatio-temporal convolution module. The feature embedding module embeds external features such as weekend holidays, geographic POIs, and weather features into the time series, the spatio-temporal attention mechanism module captures the dynamic spatio-temporal correlation of parking traffic, and the spatio-temporal convolution module captures the spatio-temporal features by using graph convolution and gated recursion units. Finally, the outputs of adjacent time series, daily series, and weekly series are weighted and fused to obtain the final prediction results, thus predicting the parking lot traffic flow more accurately and effectively. Results on real datasets demonstrate that the proposed model enhances prediction performance.

## 1. Introduction

With a steady increase in population and vehicles, traffic-related issues such as congestion and accidents have become increasingly severe. The emergence of intelligent transportation systems (ITS) provides an effective solution to these problems. Parking guidance and information (PGI) systems, especially parking flow prediction, are indispensable components of ITS. They not only offer data support and recommendations for parking management but also provide drivers with reliable parking flow predictions to plan optimal parking routes, enhance parking efficiency, locate parking spaces more quickly, and mitigate road congestion and parking-related congestion issues. Professor Donald C. Shoup from the UCLA Urban Planning School stated in “The High Cost of Free Parking,” that approximately thirty percent of vehicles on urban roads are searching for parking spaces [1]. He believes that if they could find parking spaces immediately, it could alleviate one-third of traffic congestion. Consequently, urban parking flow prediction has gained widespread attention in both academic and industrial circles.

For instance, Google Maps predicts urban parking variations based on user surveys and trajectory data, while Baidu Maps estimates real-time parking availability in cities based on contextual features such as points of interest (POIs) and map queries. Presently, navigation algorithms in maps like Amap consider real-time traffic and parking information, providing users with optimal navigation paths, including recommended parking lots. By analyzing traffic conditions, historical data, real-time traffic situations, and event information, these systems can forecast the congestion level of parking lots at specific future times, aiding users in planning parking in advance and reducing the time spent searching for parking spaces [2].

**Figure 1:**
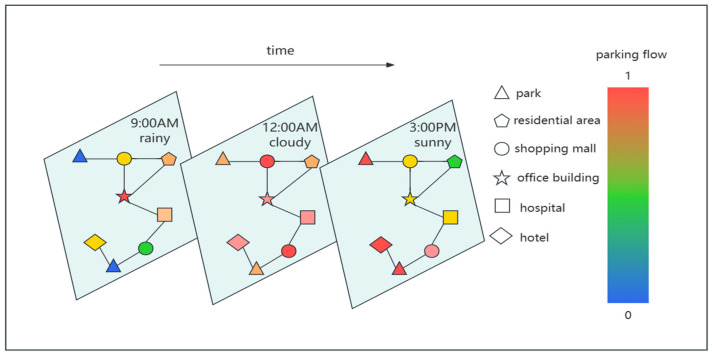
Parking lot network in a certain area.

In addition, parking information may be influenced by various external factors, such as weather conditions, emergencies, weekends, holidays, and the distribution of points of interest (POIs) near parking lots. These external factors have direct or indirect relationships with the parking flow of each parking lot. However, existing studies only focus on the impact of temporal features, such as weekends and holidays, and spatial features, such as adjacency [3]. Few studies consider the comprehensive influence of external factors such as weather conditions, weekends and holidays, parking lot adjacency, and POI information on parking information. The weather may change over time, leading to different states of parking flow under different weather conditions. Parking lots with similar POI distributions may exhibit similar trends in parking flow over time. Additionally, parking flow in individual parking lots may show different trends or periodic patterns over time, such as similar parking flow trends on Mondays but different trends on Sundays. For instance, Figure 1 depicts a temporal slice of parking lot traffic in Huangpu District, Shanghai, on 15 December 2021, a Wednesday. The solid lines delineate adjacency relationships among parking lots. Various shapes in the legend represent POIs proximate to the parking facilities, including parks, residential areas, shopping malls, office buildings, hospitals, and hotels. Commencing at 9 a.m. on the specified day, the weather conditions featured light rain. During this period, parking lot traffic at office buildings peaked, contrasting with the notably lower traffic observed on Sundays. Meanwhile, traffic volumes at parks and shopping malls remained relatively subdued. At 12 p.m., with the weather changing from light rain to cloudy, there was a rapid increase in parking lot traffic at the shopping malls. By 3 p.m., with the weather evolving from cloudy to sunny, parking lot traffic at parks and hotels reached its zenith for the day. These observations underscore that the trajectory of parking lot traffic exhibits robust spatio-temporal dependencies, coupled with correlations to weather patterns, weekends, holidays, and the distribution of nearby POIs.

However, modeling the above scenario faces the following three challenges. The first challenge is how to simulate the spatial correlation of parking lots. Parking lot traffic not only is influenced by nearby parking lots but also may be similar to traffic from distant parking lots in the same POI functional area. The second challenge is how to simulate the temporal correlation of parking lots. Parking lot traffic is related not only to adjacent time slices but also to historical time intervals, such as weekday and weekend patterns, which may exhibit certain periodicity. The third challenge is how to simulate the correlation between POIs and weather factors and parking lot traffic. Simulating parking lot traffic based solely on spatio-temporal features is insufficient. The distributions of POIs and weather conditions also have a strong relationship with parking lot traffic. To address these challenges, in this paper, we propose a spatio-temporal learning framework based on multifaceted spatio-temporal features for predicting parking lot traffic in Huangpu District, Shanghai:

- We propose a feature embedding module to model external factors influencing parking lot traffic, such as points of interest (POIs) and weather. This feature embedding module consists of an embedding layer, fusion layer, and fully connected layer. The embedding layer maps discrete categorical features (POI categories and weather types) into one-dimensional continuous vectors. Subsequently, the fusion layer concatenates the embedding features with the model’s input features. Finally, the fully connected layer maps the embedded representations to hidden dimensions, transforming the composite input features into higher-dimensional feature representations using weight matrices and activation functions.
- We construct a spatio-temporal attention mechanism to learn the dynamic spatio-temporal correlations of parking lot occupancy data. In this mechanism, the spatial attention mechanism models the complex spatial correlations between different parking lots to identify the degree of correlation between different locations and adjust the model’s predictions accordingly. Meanwhile, the temporal attention mechanism captures the dynamic temporal autocorrelations between different time intervals to capture the changing trends of parking lot occupancy over time.
- We construct a spatio-temporal convolutional module to capture the spatio-temporal features of parking lot traffic. We utilize graph convolutions and convolutions in the temporal dimension to more accurately model and predict the spatio-temporal dependencies of parking traffic data.
- We conduct extensive experiments on a real-world parking lot traffic dataset, validating that our model outperforms existing baselines and achieves the best predictive performance.

The rest of this article is as follows. In Section 2, we introduce the work related to the prediction tasks of parking lot traffic flow. After that, a description of a parking lot traffic prediction model is given in Section 3. Section 4 describes the details and frameworks of the entire network of our model. Section 5 introduces the evaluation methods and experimental results, and this paper is summarized in Section 6.

## 2. Related Work

### 2.1. Applications of Deep Learning

Although research on traffic flow prediction has been conducted for over a decade, the advent of deep learning (DL) has ushered in a new era in this field. Currently, research on traffic flow prediction has shifted from the initial statistical and traditional machine learning models to models related to deep learning. Lv et al. (2015) proposed a novel deep learning-based traffic flow prediction method [4]. This was the first time that a deep architecture model was applied using autoencoders as building blocks to represent traffic flow features for prediction. This model utilizes stacked autoencoders (SAEs) to learn traffic flow features and is trained in a greedy layerwise fashion. Experimental results demonstrate that on the PeMS dataset, this method outperforms models such as BP neural networks, random walk prediction, and support vector machines in terms of prediction accuracy. Wu et al. (2020) [5] proposed a prediction model for the peak load of bus routes built upon the idea of the newsvendor model, which explicitly combines demand prediction with supply optimization. They devised a scaled Shepard interpolation algorithm to resolve discontinuities in the probability distribution of prediction errors arising from the new indicator. Wu et al. (2020) [6] proposed a novel scaled stacking gradient boosting decision tree (SS-GBDT) model to predict bus passenger flow with multisource datasets. It can better handle the multicollinearity issue with multisource data and prioritize the influential factors on passenger flow prediction. Li et al. (2023) [7] proposed a separate modeling approach for passenger flow prediction based on behavioral patterns. They developed a novel hybrid decision tree model coupled with a decision tree model and time series model.

### 2.2. Spatial–Temporal Forecasting

However, this study did not fully consider the impact of the spatial structure of road networks on traffic flow prediction, nor did it model and process features in both the time and spatial dimensions. Therefore, relying solely on SAEs cannot capture the various complex features in traffic flow data. Traffic flow data exhibit intricate spatio-temporal dependencies, and the patterns of data changes in the time and spatial distribution dimensions differ. Modeling only one aspect cannot achieve the desired results. Hence, researchers have begun to concurrently consider both temporal and spatial dependencies. 

For example, Jin et al. (2018) proposed spatio-temporal recurrent convolutional networks for citywide short-term crowd flows prediction (STRCNs), a model that combines convolutional neural network (CNN) and long short-term memory (LSTM) network structures to capture spatio-temporal dependencies simultaneously [8]. Experimental results on two datasets (MobileBJ and TaxiBJ) show that STRCNs outperform classical time series and other deep learning-based prediction methods. However, the study did not take into account the non-Euclidean characteristics of data in the traffic network. Subsequently, many researchers started to consider the specificity of road network structures and applied graph neural networks to traffic flow prediction to handle non-Euclidean data in traffic flow. For example, Yu et al. (2018) proposed spatio-temporal graph convolutional networks (STGCNs), a model that combines temporal and spatial models [9]. Instead of applying regular convolutional and recurrent units, it formulates the problem on graphs and builds the model with complete convolutional structures, which enables a much faster training speed with fewer parameters. Experimental results on two datasets, BJER4 and PeMSD7, show that the STGCN outperforms other deep models. Li et al. (2018) proposed the diffusion convolutional recurrent neural network (DCRNN), a deep learning framework for traffic forecasting that incorporates both spatial and temporal dependency in the traffic flow [10]. DCRNN captures the spatial dependency using bidirectional random walks on the graph and the temporal dependency using the encoder–decoder architecture with scheduled sampling. Zhao et al. (2019) proposed a temporal graph convolutional network (T-GCN) model, which combines the graph convolutional network (GCN) and the gated recurrent unit (GRU) to capture spatial and temporal dependencies, respectively [11]. The GCN is used to learn complex topological structures for capturing spatial dependence and the gated recurrent unit is used to learn dynamic changes in traffic data for capturing temporal dependence.

Due to inherent issues in the composition of recurrent neural network (RNN) models, their sequential computation mode affects parallel computing and the improvement of training speed. Therefore, in 2017, the Google team led by Vaswani proposed the Transformer model. This model abandons the use of neural network units with recurrent recursive structures and instead employs a multihead attention mechanism to learn features between word vectors. Compared to the original RNN models and their variants, the Transformer model has made significant strides in many daily tasks in natural language processing. Inspired by the success of the Transformer model in the field of natural language processing, many studies in the traffic flow prediction domain have also begun to adopt attention models. Zheng et al. captured spatio-temporal dependencies by utilizing spatio-temporal embeddings and attention mechanisms [12]. They devised a gate fusion module to amalgamate the output results of time attention and space attention. Subsequently, they formulated a model with an encoder–decoder structure based on the spatio-temporal attention module and designed a trans-attention module to manage the input–output interaction between the encoder and decoder, thereby minimizing errors. 

Guo et al. introduced the ASTGCN model, which incorporated a spatio-temporal attention mechanism to comprehend the dynamic spatio-temporal correlations within traffic data [13]. Additionally, they innovated a novel spatio-temporal convolution module, encompassing graph convolution for extracting spatial features from the traffic network and time convolution for capturing dependencies from adjacent time slices. Acknowledging that various types of temporal dependencies may differ, they employed three distinct traffic flow prediction models to capture dependencies at the levels of proximity, daily patterns, and weekly trends, subsequently consolidating the final results.

### 2.3. The Influence of External Factors

Although in traffic flow prediction the data flow itself and network topology are the most significant influencing factors, the impact of many other factors may also alter the patterns of traffic flow changes. Different short- and long-term dependencies considered by ASTGCN are one example. Additionally, factors such as weather and temperature are also crucial influencing factors. Zhang et al. (2017) proposed a method for predicting pedestrian flow using residual networks, considering the impact of external factors such as weather and holiday events beyond flow data [14]. They designed an end-to-end structure of an ST-ResNet based on unique properties of spatio-temporal data and employed the residual neural network framework to model the temporal closeness, period, and trend properties of crowd traffic. In 2019, Chen Zehao considered the impact of factors such as weather, time, and cycles on traffic flow prediction in his research. Zhu et al. (2023) designed a knowledge graph for traffic prediction [15]. They integrated the knowledge obtained from knowledge graph embedding methods into spatio-temporal graph convolutional networks using KF-Cell. However, they overlooked the temporal dependencies of the model at each hour, day, and week.

### 2.4. Parking lot Traffic Flow Prediction

Zhang et al. (2019) proposed a semi-supervised spatio-temporal learning framework that combines environmental contextual factors with sparse real-time parking availability data [16]. This framework first proposed a hierarchical graph convolution structure to model non-Euclidean spatial autocorrelation among parking lots. It proposed a contextual graph convolution block and a soft clustering graph convolution block to capture local and global spatial dependencies between parking lots. The authors considered global spatial correlations for node-level prediction and used them for citywide parking availability prediction. Finally, they adopted a recurrent neural network to incorporate dynamic temporal dependencies of parking lots [17]. Zeng et al. (2022) proposed a stacked GRU-LSTM model for parking occupancy prediction. It combined GRU’s advantage in prediction efficiency with LSTM’s advantage in prediction accuracy and took into account multifactors, e.g., occupancy, weather conditions, and vacations [18]. Zheng et al. (2020) studied short-term parking demand prediction. Focusing on the regular pattern of the distribution of typical parking arrivals and departures, they constructed a parking demand prediction model utilizing the Markov birth and death process. They calibrated model parameters utilizing a curve fitting method and undetermined coefficients method [19]. Mufida et al. (2023) proposed a novel two-step clustering technique that grouped parking lots based on their spatio-temporal patterns [20].

In the above research on parking lot flow prediction, there are some careless considerations in the complex and irregular spatial relationship modeling of parking lots, including the time autocorrelation relationship modeling of parking lot flow and the characteristics modeling of POIs of parking lots and weather, weekends, and holidays. Based on this, this paper undertakes deep research on how to integrate these problems and make better modeling to accurately predict the parking lot flow.

## 3. Preliminaries

### 3.1. Problem Definition

First of all, we define the parking lot network as an undirected graph (G=(V, E, A), as shown in Figure 1, where V is a finite set of nodes with $\left|V\right|=N$ nodes; E is a set of edges representing the connectivity between nodes; and $A\in R^{N\times N}$ denotes the adjacency matrix of graph G.

In the parking lot network, each node records multiple features, with the f-th feature being the parking lot’s flow, where $f\in \left(1,\;\ldots ,F\right)$. Let $x_{tn}\in R$ represent all features of node n at time t.

$X_{t}={\left(x_{1t},x_{2t},\ldots ,x_{Nt}\right)}^{T}\in R^{N\times F}$ represents the values of all features of all nodes at time t, where N is the number of nodes and F is the number of features. $X={\left(X_{1},X_{2},\ldots ,X_{\tau }\right)}^{T}\in R^{N\times F\times \tau }$ represents the values of all features of all nodes over τ time slices, where τ is the length of the time series. Additionally, we set $y_{nt}=x_{fn}\in R\;$to represent the parking lot flow of node n at future time t.

Given X, predict the future parking lot flow $\;Y=\;{\left(y_{1},\;y_{2},\;\ldots \;,\;y_{n}\right)}^{T}\;\in R^{N\times T_{p}}$ all nodes in the entire parking lot network for the next $T_{p}$ time slices.$y_{n}=\left(y_{n,\tau +1},y_{n,\tau +2},\ldots ,y_{n,\tau +T_{p}}\right)\in R^{T_{p}}$ represents the future parking lot network of node n from τ+1 [21].

### 3.2. Data Pre-Processing

In order to facilitate the modeling, we need to aggregate the parking data of the parking lot every 15 min, a total of 96 time slices per day, and calculate the parking flow of the parking lot for each time slice. Then the weather information collected from the weather website is divided into five levels according to the traffic meteorological index. Then, the POI information of each parking lot collected from AmAP is divided into six labels: park, residential area, shopping mall, office building, hospital, and hotel. The latitude and longitude information is used to calculate the distance between each parking lot according to the Haversine formula. The distance threshold of 1.5 km is set, which is 1 within the threshold and 0 beyond the threshold to generate the adjacency matrix. The purpose is to study the spatio-temporal and external characteristics of parking flow in parking lots and provide richer knowledge for parking flow prediction in parking lots.

## 4. Proposed Method

**Figure 2:**
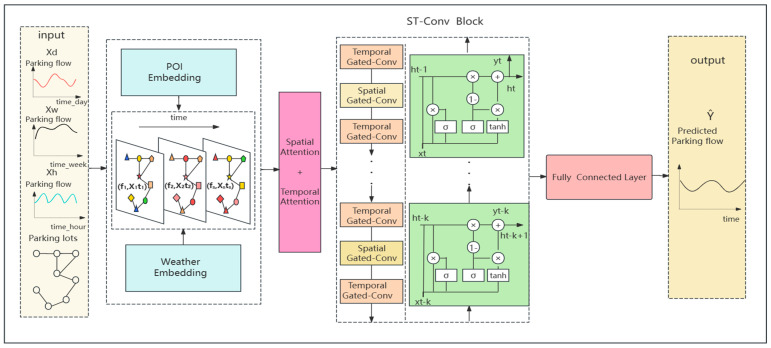
The framework of MFF-STGCN. POI Embedding: the embedding of points of interest near the parking lot; Weather Embedding: the embedding of weather information; GCN: Graph Convolution; Conv: convolution; FC: fully connected; ST block: spatial-temporal block.

In this part, we describe our proposed model, parking lot traffic prediction model based on the fusion of multifaceted spatio-temporal features (MFF-STGCN), which is shown in Figure 2. The model consists of a feature embedding module, a spatio-temporal attention mechanism module, and a spatio-temporal convolution module.

The feature embedding module embeds external features such as weekend holidays, geographic POIs, and weather features into the time series. It includes an embedding layer, fusion layer, and fully connected layer. The embedding layer maps discrete categorical features (POI categories and weather types) into one-dimensional continuous vectors.

The spatio-temporal attention mechanism module captures the dynamic spatio-temporal correlation of parking traffic. It consists of spatial and temporal attention layers. The spatial attention layer and temporal attention layer calculate spatial attention scores. Sigmoid is used to generate attention scores, and softmax normalizes the scores.

The spatio-temporal convolution module captures the spatio-temporal features by using graph convolution and gated recursion units. It consists of graph convolutional layers and temporal convolutional layers. ReLU activation is used after the convolution operation.

Finally, the outputs of adjacent time series, daily series, and weekly series are weighted and fused to obtain the final prediction results, thus predicting the parking lot traffic flow more accurately and effectively.

### 4.1. Inputs for Daily, Hourly, and Weekly Modules

**Figure 3:**
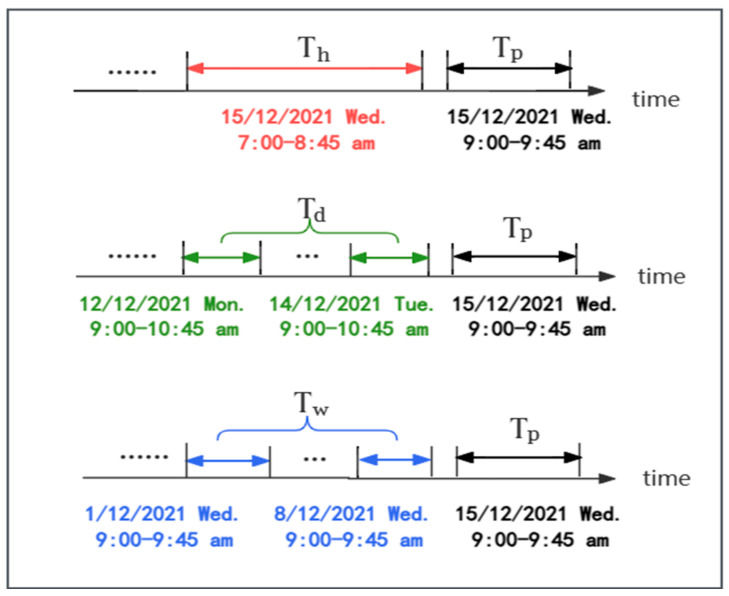
An example of constructing the input of time series segments (suppose the size of the predicting window is three-quarters of an hour). $T_{h}$, $T_{d}$, and $T_{w}$ are twice the value of $T_{p}$.

Suppose the sampling frequency is s times per day. Assume the current time is $t_{0}$, and the size of the prediction window is $T_{p}$. As shown in Figure 3, we extract three time series segments along the time axis with lengths $T_{h}$, $T_{d}$, and $T_{w}\;$as inputs for the recent, daily periodic, and weekly periodic components, respectively, where $T_{h}$, $T_{d}$, and $T_{w}$ are multiples of $T_{p}$. The details of the recent segment are shown as follows:

(1) $$X_{h}=\left(X_{t_{0}-T_{h}+1},X_{t_{0}-T_{h}+2},\ldots ,X_{t_{0}}\right)\in R^{N\times F\times T_{h}}$$

where $X_{h}$ is a segment of the historical time series directly adjacent to the prediction period, as shown in the red part of Figure 3. Therefore, the recently passed parking lot flow inevitably affects the future parking lot flow. The details of the daily period segment are shown as follows:

(2) $$X_{d}=(X_{t_{0}-T_{d}/T_{p}\cdot s+1},\ldots ,X_{t_{0}-T_{d}/T_{p}\cdot s+T_{p}},\;X_{t_{0}-\left(T_{d}/T_{p}-1\right)\cdot s+1},\ldots ,$$

$$X_{t_{0}-\left(T_{d}/T_{p}-1\right)\cdot s+T_{p}}\;,\ldots ,X_{t_{0}-s+1},\ldots ,X_{t_{0}-s+T_{p}})\in R^{N\times F\times T_{d}}$$

where $X_{d\;}$is a segment of the past few days identical to the prediction period, as shown in the green part of Figure 3. Due to people’s daily routines, parking lot data may exhibit repetitive patterns, such as morning peaks every day. The purpose of the daily periodic component is to simulate the daily cycle of parking lot data. The details of the weekly period segment are shown as follows:

(3) $$X_{w}=(X_{t_{0}-{7\cdot T}_{w}/T_{p}\cdot s+1},\ldots ,X_{t_{0}-{7\cdot T}_{w}/T_{p}\cdot s+T_{p}},X_{t_{0}-7\cdot \left(T_{w}/T_{p}-1\right)\cdot s+1},\ldots ,$$

$$\;\;\;X_{t_{0}-7\cdot \left(T_{w}/T_{p}-1\right)\cdot s+T_{p}}\;,\ldots ,X_{t_{0}-7\cdot s+1},\ldots ,X_{t_{0}-7\cdot s+T_{p}})\;\in R^{N\times F\times T_{w}}$$

where $X_{w}\;$is a segment of the past several weeks identical to the prediction period, as shown in the blue part of Figure 3. Due to the weekly cycle of human activities, parking lot data may exhibit periodic patterns repeating every week. The purpose of the weekly periodic component is to simulate the weekly cycle of parking lot data.

Thus, if predicting the parking lot flow for Wednesday morning, 15 December 2021, from 9:00 to 9:45, the model uses the parking lot flow from 7:00 to 8:45 on the same day as input for the hourly model, the parking lot flow from 9:00 to 9:45 on the previous two days (Monday, 13 December and Tuesday, 14 December) as input for the daily model, and the parking lot flow from 9:00 to 9:45 on the previous two Wednesdays (1 December and 8 December) as input for the weekly model. The model leverages these three dimensions of parking lot flow to jointly predict future parking lot flow.

So, this issue can be expressed as

(4) $$\;X_{t}=H_{\theta }\cdot \left(X_{h},X_{d},X_{w},X_{wea},X_{poi};G\right)$$

where $H_{\theta }$ is the prediction model that uniformly models past parking lot flow $X_{h},X_{d},X_{w}$, weather feature $X_{wea},\;$ and POI information$\;X_{poi}$ and fully integrates multiple features. It captures the spatio-temporal relationships and external information characteristics of parking lots and predicts the parking lot flow $X_{t}\;$at a future time [22].

### 4.2. Fusion of Flow Features and External Features

According to the periodicity of parking traffic, the time characteristics of parking traffic are extracted from three dimensions: hour, day, and week. The same network structure is used in each dimension. In the previous section, we obtained hourly period component $X_{h}$, daily period component $X_{d}$, and weekly period component $X_{w}$. We fuse the parking lot’s POI features $X_{poi}\;and\;$weather features$\;X_{wea}$ using an additive method to create $x_{c}^{h}$. We then use a multilayer perceptron (MLP) to extract features and reshape them to match the vector dimension of the hourly period component $X_{h}$ while keeping the daily period and weekly period components the same, obtaining the feature $x_{c}^{d},x_{c}^{w}$. Finally, the fusion features $X_{c}\;$are as follows [23]:

(5) $$x_{c}^{h}={\mathrm{conv}}_{\theta }\cdot \left(X_{wea}+X_{poi}+X_{h}\right)$$

(6) $$x_{c}^{d}={\mathrm{conv}}_{\theta }\cdot \left(X_{wea}+X_{poi}+X_{d}\right)$$

(7) $$\;x_{c}^{w}={\mathrm{conv}}_{\theta }\cdot \left(X_{wea}+X_{poi}+X_{w}\right)$$

(8) $$\;X_{c}=G_{\theta }\cdot \left(x_{c}^{h},x_{c}^{d},x_{c}^{w};G\right)$$

### 4.3. Spatial-Temporal Attention

Our model introduces a novel spatial-temporal attention mechanism designed to capture the dynamic spatial and temporal correlations within the parking lot network. This mechanism incorporates two types of attention: spatial attention and temporal attention.

In the spatial domain, interactions between parking lot conditions at various locations are highly dynamic. To address this, we employ an attention mechanism to dynamically capture the correlations among nodes in the spatial dimension.

Take the spatial attention in the daily period component as an example:

(9) $$S=V_{s}\cdot \sigma \left(\left(X_{d}^{\left(k-1\right)}W_{1}\right)W_{2}{\left(W_{3}X_{d}^{\left(k-1\right)}\right)}^{T}+b_{s}\right)$$

(10) $$\;S_{m,n}^{\prime }=\frac{\exp\left(S_{m,n}\right)}{\sum_{n=1}^{N}exp\left(S_{m,n}\right)}\;\;\;\;\;\;\;\;\;$$

where $X_{d}^{\left(k-1\right)}=\left(X_{1},X_{2},\ldots ,X_{T_{k-1}}\right)$ is the input of the k th spatial-temporal block, belonging to $R^{N\times C_{k-1}\times T_{k-1}}$. $C_{k-1}$ is the number of channels of the input data in the k th layer. When k=1 , $C_{0}=F$, $T_{k-1}$ is the length of the temporal dimension in the k th layer. When k=1 , in the daily period component $T_{0}=T_{d},\;$in the hourly period component $T_{0}=T_{h}$, and in the weekly period component $T_{0}=T_{w}$. In Equation (9), $V_{s}$, $b_{s}\in R^{N\times N}$, $W_{1}\in R^{T_{k-1}}$, $W_{2}\in R^{Ck-1\times T_{k-1}}$, $W_{3}\in R^{Ck-1}$ are learnable parameters, and sigmoid σ is used as the activation function. The attention matrix S is dynamically computed according to the current input of this layer. The value of an element $S_{m,n}\;in\;S$ semantically represents the correlation strength between node m and node n. Then a softmax function is used to ensure the attention weights of a node sum to one. When performing the graph convolutions, we accompany the adjacency matrix A with the spatial attention matrix $S^{\prime }\in R^{N\times N}$ to dynamically adjust the impacting weights between nodes [24].

In the temporal dimension, parking lot traffic conditions vary dynamically across different periods and interact with each other. To address this, our model employs the attention mechanism to dynamically capture the correlations between nodes in the temporal dimension.

(11) $$E=V_{e}\cdot \sigma \left(\left({\left(X_{k-1}^{\left(d\right)}\right)}^{T}U_{1}\right)U_{2}\left(U_{3}X_{k-1}^{\left(d\right)}\right)+b_{e}\right)$$

(12) $$E_{m,n}^{\prime }=\frac{\exp\left(E_{m,n}\right)}{\sum_{n=1}^{T_{k-1}}\exp\left(E_{m,n}\right)}$$

where $V_{e}$, $b_{e}\in R^{T_{k-1}\times T_{k-1}}$, $U_{1}\in R^{N}$,$U_{2}\in R^{Ck-1\times N}$, $U_{3}\in R^{Ck-1}$ are learnable parameters. The temporal correlation matrix E is determined by the varying inputs. The value of an element $E_{m,n}\;in\;E$ semantically indicates the strength of dependencies between time m and n. At last, E is normalized by the softmax function. We directly apply the normalized temporal attention matrix to the input and obtain ${\hat{X}}_{k-1}^{\left(d\right)}=\left({\hat{X}}_{1},{\hat{X}}_{2},\ldots ,{\hat{X}}_{T_{k-1}}\right)=\left(X_{1},X_{2},\ldots ,X_{T_{k-1}}\right)E^{\prime }\in R^{N\times Ck-1\times T_{k-1}}$ to dynamically adjust the input by merging relevant information [25].

### 4.4. Spatial–Temporal Convolution 

The spatial–temporal attention module enables the network to automatically allocate more attention to pertinent information. The input, adjusted by the attention mechanism, is then passed to the spatial–temporal convolution module. This module comprises a graph convolution in the spatial dimension, which captures spatial dependencies from neighboring nodes, and a convolution along the temporal dimension, which exploits temporal dependencies from nearby time steps [26].

Spectral graph theory extends the convolution operation from grid-based data to graph-structured data. The parking lot network is inherently represented as a graph structure, where the features of each node can be interpreted as signals on the graph. Therefore, to fully leverage the topological properties of the parking lot network, we employ graph convolutions based on spectral graph theory at each time slice to directly process these signals. This approach exploits signal correlations on the parking lot network in the spatial dimension. The spectral method transforms a graph into an algebraic form, enabling analysis of topological attributes such as connectivity within the graph structure [27].

In spectral graph analysis, a graph is represented by its corresponding Laplacian matrix. The properties of the graph structure can be obtained by analyzing the Laplacian matrix and its eigenvalues. The Laplacian matrix of a graph is defined as L=D−A, and its normalized form is $L=I_{N}-D^{-\frac{1}{2}}AD^{-\frac{1}{2}}\in R^{N\times N}$, where A is the adjacency matrix, $I_{N}$ is a unit matrix, and the degree matrix $D\in R^{N\times N}$ is a diagonal matrix consisting of node degrees, $D_{mm}=\sum_{n}A_{mn}$.

The eigenvalue decomposition of the Laplacian matrix is $L=U\Lambda U^{T}$, where $\Lambda \;=\;\mathrm{diag}\left(\left[\lambda_{0},\ldots ,\;\lambda_{N-1}\right]\right)\in R^{N\times N}\;$is a diagonal matrix, and U is the Fourier basis. Taking the parking lot flow at time t as an example, the signal all over the graph is $x=x_{f_{t}}\in R^{N},$ and the graph Fourier transform of the signal is defined as $\hat{x}=U^{T}x.$ According to the properties of the Laplacian matrix, U is an orthogonal matrix, so the corresponding inverse Fourier transform is $x=U\hat{x}$. Graph convolution is a convolution operation implemented by using linear operators that diagonalize in the Fourier domain to replace the classical convolution operator. Based on this, the signal x on the graph G is filtered by a kernel $g_{\theta }$:

(13) $$\;g_{\theta }*G_{x}=g_{\theta }\left(L\right)x=g_{\theta }\left(U\Lambda U^{T}\right)x=Ug_{\theta }\left(\Lambda \right)U^{T}x$$

where *G represents a graph convolution operation. Since the convolution operation of the graph signal is equal to the product of these signals which have been transformed into the spectral domain by the graph Fourier transform, the above formula can be understood as Fourier transforming $g_{\theta }\;$and x, respectively, into the spectral domain, then multiplying their transformed results and performing the inverse Fourier transform to obtain the final result of the convolution operation. However, it is expensive to directly perform the eigenvalue decomposition on the Laplacian matrix when the scale of the graph is large [28]. Therefore, this paper adopts Chebyshev polynomials to approximate the eigenvalue decomposition efficiently:

(14) $$g_{\theta }*G_{x}=g_{\theta }\left(L\right)x=\sum_{k=0}^{K-1}\theta_{k}T_{k}\left(\tilde{L}\right)x$$

(15) $$\tilde{L}=\frac{2}{\lambda_{\max}}L-I_{N}$$

where the parameter $\theta \in R^{K}$ is a vector of polynomial coefficients, where $\lambda_{\max}\;$is the maximum eigenvalue of the Laplacian matrix. The recursive definition of the Chebyshev polynomial is $T_{k}\left(x\right)=2xT_{k-1}\left(x\right)-T_{k-2}\left(x\right)$,where $T_{0}\left(x\right)=1,\;T_{1}\left(x\right)=x.$ Using the approximate expansion of the Chebyshev polynomial to solve this formulation corresponds to extracting information of the surrounding 0- to ${\left(K-1\right)}^{th}$-order neighbors centered on each node in the graph by the convolution kernel $g_{\theta }$. The graph convolution module uses the rectified linear unit (ReLU) as the final activation function, i.e., $\mathrm{ReLU}\left(g_{\theta }*G_{x}\right)$.

To dynamically adjust the correlations between nodes, for each term of the Chebyshev polynomial, we accompany $T_{k}\left(\tilde{L}\right)\;$with the spatial attention matrix $S^{\prime }\in R^{N\times N}$, then obtain $T_{k}\left(\tilde{L}\right)\;⊙S^{\prime }\;$, where ⊙ is the Hadamard product. Therefore, the above graph convolution formula changes to $g_{\theta }*G_{x}=g_{\theta }\left(L\right)x=\sum_{k=0}^{K-1}\theta_{k}\left(T_{k}\left(\tilde{L}\right)⊙S^{\prime }\right)x$.

We can generalize this definition to the graph signal with multiple channels. In the daily period component, the input is ${\hat{X}}_{d}^{\left(k-1\right)}=\left({\hat{X}}_{1},{\hat{X}}_{2},\ldots ,{\hat{X}}_{T}^{\left(k-1\right)}\right)\in R^{N\times C^{\left(k-1\right)}\times T^{\left(k-1\right)}}$, where the feature of each node has $C^{\left(k-1\right)}$ channels. For each time slice t, performing $C_{r}\;$ filters on the graph ${\hat{X}}_{t}$, we obtain $g_{\theta }-G{\hat{X}}_{t},$ where $\Theta =\left(\Theta_{1},\Theta_{2},\ldots ,\Theta_{C_{k}}\right)\in R^{K\times C^{\left(k-1\right)}\times C_{k}}$ is the convolution kernel parameter. Therefore, each node is updated by the information of the 0 to K−1 neighbors of the node [29].

After the graph convolution operations have captured neighboring information for each node on the graph in the spatial dimension, a standard convolution layer in the temporal dimension is further stacked to update the signal of a node by integrating the information from neighboring time slices. Also, take the operation on the *k*th layer in the recent component as an example:

(16) $$X_{d}^{\left(k\right)}=\mathrm{ReLU}\left(\Phi *\left(\mathrm{ReLU}\left(g_{\theta }*GX_{d}^{\left(k-1\right)}\right)\right)\right)\in R^{Ck\times N\times T_{k}}$$

where * denotes a standard convolution operation, Φ is the parameters of the temporal dimension convolution kernel, and the activation function is ReLU.

In conclusion, the spatial–temporal convolution module effectively captures the temporal and spatial features of parking lot traffic data. This module, along with the spatial–temporal attention module, forms a spatial–temporal block. Multiple spatial–temporal blocks are stacked to extract a broader range of dynamic spatial–temporal correlations. Finally, a fully connected layer is added to ensure that the output of each component matches the dimension and shape of the forecasting target. ReLU is used as the activation function in the final fully connected layer.

### 4.5. Multicomponent Fusion

In this section, we explore the integration of the outputs from the three components. Taking the example of forecasting parking lot flow across the entire parking lot network at 9:00 a.m. on Wednesday, we observe that certain areas experience peak parking lot traffic periods in the morning, making the outputs of the daily period and weekly period components more significant. However, in other areas, distinct parking lot traffic cycle patterns may not be present, reducing the influence of the daily period and weekly period components [30]. Consequently, when combining the outputs of different components, the weights of the three components for each node vary and should be learned from historical data. Therefore, the final prediction result after fusion is as follows:

(17) $$Y=W_{h}⊙{\hat{Y}}_{h}+W_{d}⊙{\hat{Y}}_{d}+W_{w}⊙{\hat{Y}}_{w}$$

where ⊙ is the Hadamard product, and $W_{h}$, $W_{d}$, and $W_{w}$ are learning parameters, reflecting the influence degrees of the three temporal-dimensional components on the forecasting target. ${\hat{Y}}_{h}$, ${\hat{Y}}_{d}$, and ${\hat{Y}}_{w}$ are outputs of hourly, daily, and weekly modules.

## 5. Results

In this section, to evaluate the performance of our model, we carried out experiments on a real-world parking lot traffic dataset. First, we introduced datasets, baseline models, assessment measures, and parameter settings. Then, our model was compared to the baseline model, and the impact of different strategies was analyzed. Finally, we present experimental results and model effect analysis under different constraints.

### 5.1. Experiments Settings

**Figure 4:**
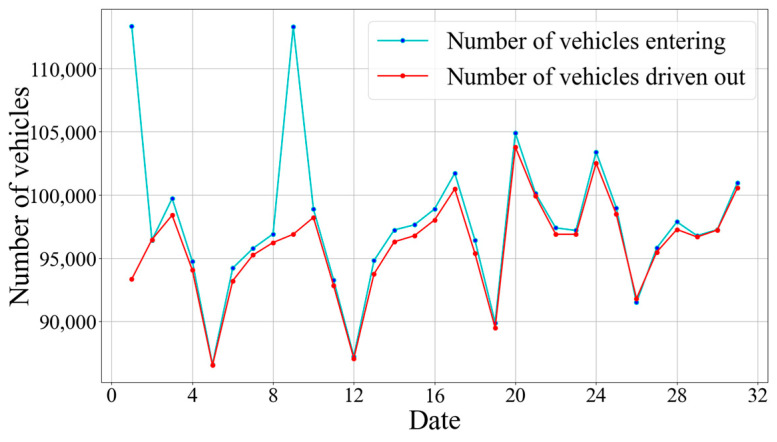
Daily inbound and outbound vehicle statistics.

**Datasets**: Although there are several open parking lot flow datasets, it is not easy to collect parking lot data and additional knowledge information including POI data and weather data of the same area for the same period together. Limited by the data acquisition and the difficulty of constructing the knowledge graph, the experiments in this paper are all based on one dataset from Huangpu District, Shanghai. However, due to the generality of our experimental setup, experiments can be easily validated in other cities as long as the dataset is given. We used a real dataset from the large city of Shanghai, China. The dataset covers the period from 1 December 2021, to 30 December 2021. All parking records are captured every 15 min from a publicly accessible application, where parking occupancy information is collected in real time by sensors. We associated points of interest (POIs) and weather conditions with each parking lot and aggregated the registration records near each parking lot every 15 min as data. POI and check-in data were collected through the Gaode Map location API, while current weather conditions were obtained from the China Weather Network. We sorted the data in chronological order, with the first thirty percent used as the training set, the next twenty percent for validation, and the remaining for testing. Summary statistics of the dataset are provided in Figure 4.

The mathematical software used included Python3.10, utilizing the NumPy library for vector and matrix operations, the Pandas library for data processing and time series analysis, and the PyTorch2.2.0 framework for tensor operations and constructing deep learning models. We tested the number of terms of Chebyshev polynomial K ∈{1, 2, 3}. Considering the computing efficiency and the degree of improvement of the forecasting performance, we set K=3 and the kernel size along the temporal dimension to 3. In our model, both graph convolutional layers and temporal convolutional layers employ 64 convolutional kernels, and the period of the data is adjusted by controlling the step size of the temporal convolutions. ReLU activation is used after the convolution operation. For the lengths of the three segments, we set them as $T_{h}=8$, $T_{d}=4$, $T_{w}=8$. The size of the predicting window $T_{p}=3$, that is to say, we aimed at predicting the parking lot flow over three-quarters of an hour in the future. In this paper, the mean absolute error (MAE) between the estimator and the ground truth is used as the loss function and minimized by backpropagation. During the training phase, the batch size is 64 and the learning rate is 0.0001.

**Baseline:** We compared our model with several existing approaches. The introductions of those methods are shown below.

**LSTM:** Long short-term memory network, a special RNN model [31].

**GRU:** Gated recurrent unit network, a special RNN model [32].

**STGCN**: A graph neural network model for traffic forecasting. It models both spatial and temporal dependency with convolution structure. The input graph is constructed as described in the original paper but keeps the same graph connectivity with our CxtConv [33].

**SST-GNN:** Simplified spatio-temporal traffic forecasting model using a graph neural network [34].

**ASTGCN:** Attention-based spatial-temporal graph convolutional network for traffic flow forecasting [13].

**Metrics:** To measure and evaluate the performance of different methods, mean absolute errors (MAEs) and root mean squared errors (RMSEs) are adopted.

### 5.2. Performance Comparison

We compare our models with six baseline methods using the dataset from Huangpu District, Shanghai. Table 1 presents the average results of parking lot traffic flow prediction performance over three-quarters of an hour.

Generally, as the prediction interval for parking lot traffic increases, forecasting becomes more challenging, and prediction errors also increase. When considering only temporal correlations, traditional LSTM and GRU models perform well in short-term forecasting. However, their accuracy declines noticeably as the prediction horizon expands, demonstrating their limited ability to model nonlinear and complex parking lot traffic data. In contrast, methods based on deep learning generally achieve better prediction results than traditional time series analysis methods. Among them, SSTGCN outperforms GRU and LSTM, suggesting that hourly, daily, and weekly parking lot flow patterns are better captured. This is because parking lot traffic exhibits temporal correlations such as daily and weekly patterns. ASTGCN and STGCN outperform LSTM and GRU, indicating that parking lot data show certain spatial correlations; adjacent parking lots exhibit similar traffic patterns spatially. ASTGCN outperforms STGCN, highlighting that the spatio-temporal attention mechanism effectively captures dynamically changing parking lot traffic data.

Finally, our proposed MFF-STGCN model achieves the highest prediction accuracy and shows slower degradation with increasing prediction intervals. This model not only considers temporal and spatial correlations in parking lot traffic data but also integrates external features such as points of interest (POIs) and weather conditions. Parking lot traffic is influenced by specific POI categories in its vicinity; parking lots in similar POI functional zones tend to have similar traffic volumes. Additionally, different weather conditions have varying effects on parking lot traffic.

**Figure 5:**
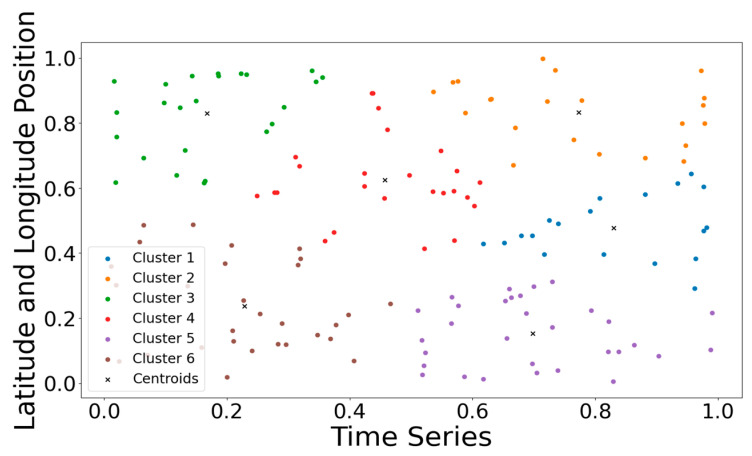
Parking lot cluster visualization.

Figure 5 shows the k-means clustering of all parking lots in the Huangpu District of Shanghai. The average parking flow at the same time of each day is taken as the time feature of this time, and the time feature of each parking lot is composed of the average flow at all times. The longitude and latitude coordinates of the parking lot are used as the spatial features, and the temporal and spatial characteristics of the parking lot are combined to cluster. The silhouette coefficient is used to determine the number of categories, and six clusters are obtained. The experimental results also confirm the interpretability and accuracy of our classification of POI function areas in the parking lot.

**Figure 6:**
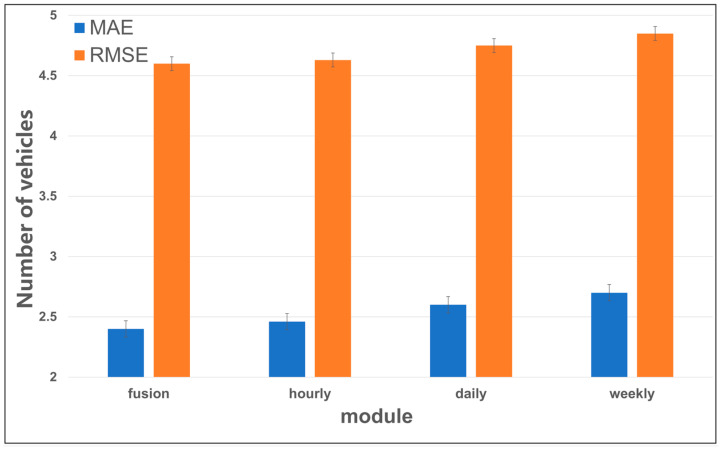
Comparison of module prediction accuracy.

Figure 6 shows the comparison between the independent prediction accuracy of the three dimensions hourly, daily, and weekly modules in the MFF-STGCN model and the prediction accuracy of the model after the weighted fusion of the three modules, from which it can be seen that the prediction accuracy of the weighted fused model is higher than that of the independent prediction of the individual modules, indicating that the fusion of the model’s information in the different dimensions improves the accuracy of the parking lot flow prediction. Due to the daily, weekly, and temporal similarities in parking traffic, overall accuracy in daily, weekly, and hourly modules of prediction was good. Subsequently, we conducted multiple dataset partitioning experiments and found that in some parking lots, the accuracy of daily and weekly modules decreased, showing a disparity compared to the accuracy of the weighted fusion model. This suggests that some parking lots may lack distinct traffic cycle patterns. Therefore, by learning influence weights from historical data and weighting the fused outputs of different components, the weighted fusion model demonstrates higher and more stable prediction accuracy.

**Figure 7:**
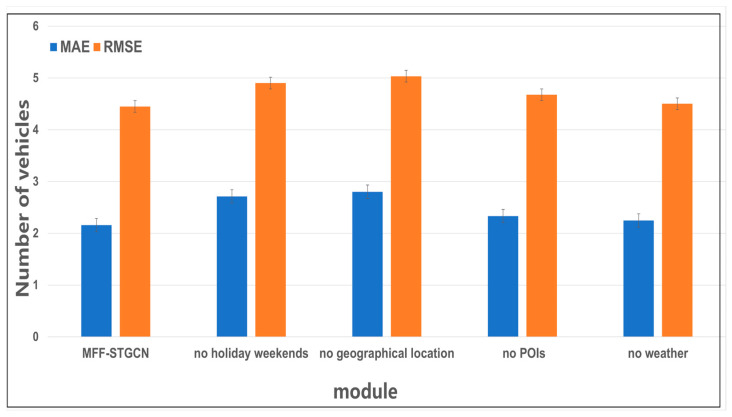
Comparison of feature prediction accuracy.

Figure 7 shows the comparison of feature prediction accuracy. We successively removed the weekend holidays, daily, and weekly modules, geographic location module, POI feature embedding module, and weather feature embedding module. Specifically, removing the geographic location module involved setting the adjacency matrix generated from latitude and longitude of each parking lot to either an all-ones matrix or an all-zeros matrix and conducting multiple experiments. Subsequently, we incrementally reintroduced each of these modules and repeated the experiments. According to Figure 7, holiday weekends, geographic location, POIs, and weather all have varying degrees of impact on predicting parking lot traffic flow. Firstly, parking lot traffic flow exhibits temporal correlations such as daily and weekly patterns. Parking lot traffic on weekdays shows similarities with traffic on previous days and weeks. There are differences between weekday and weekend traffic; for example, parking lots near office buildings experience higher traffic on weekdays and lower traffic on weekends. Secondly, parking lot traffic exhibits spatial correlations. Parking lots in close proximity show similar traffic patterns. Thirdly, parking lot traffic is influenced by the surrounding POI areas. Parking lots in similar POI functional zones exhibit similar traffic trends, even if they are geographically distant. For example, parking lots near office buildings experience higher traffic on weekdays, while parking lots near shopping malls or parks experience lower traffic. Finally, parking lot traffic is affected by weather conditions. For instance, in residential areas, worse weather conditions lead to higher traffic meteorological indices and lower parking lot traffic volumes. Our MFF-STGCN model considers all four features comprehensively and achieves more accurate predictions of parking lot traffic flow.

**Figure 8:**
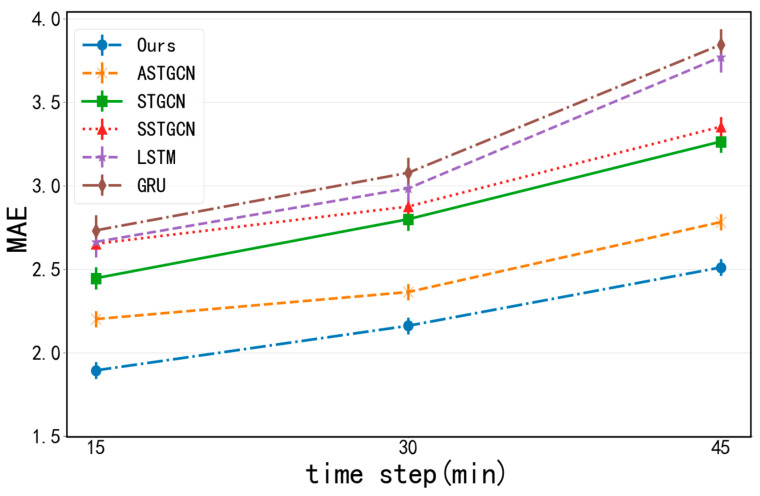
The prediction results with MAE as an evaluation index of different methods.

**Figure 9:**
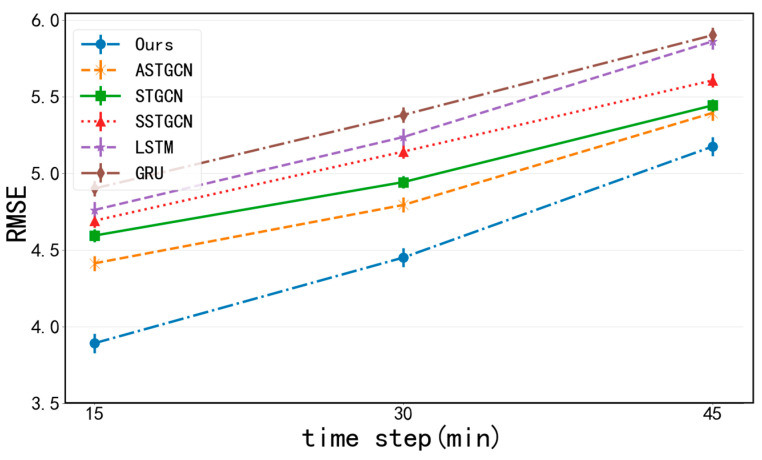
The prediction results with RMSE as an evaluation index of different methods.

Figure 8 and Figure 9 show the changes in the prediction performance of various methods as the prediction interval increases. Overall, as the prediction interval becomes longer and the corresponding prediction difficulty becomes greater; hence, the prediction errors also increase. As can be seen from the figures, the methods only taking the temporal correlation into account can achieve good results in short-term prediction. In contrast, the performance of ASTGCN declines at a slower rate than these methods. This is mainly due to the fact that ASTGCN can simultaneously take into account spatio-temporal correlations, which are more important in long-term forecasting. The errors of deep learning methods increase slowly with prediction interval increases, and their overall performance is good.

Our MFF-STGCN model achieves the best prediction performance almost all the time. Especially in long-term prediction, the differences between MFF-STGCN and other baselines are more significant, showing that combining features such as weather conditions, weekends, and POI distribution embedding with a spatio-temporal attention mechanism, graph convolution, and temporal convolution mechanism can better capture the dynamic spatio-temporal patterns of traffic data.

**Figure 10:**
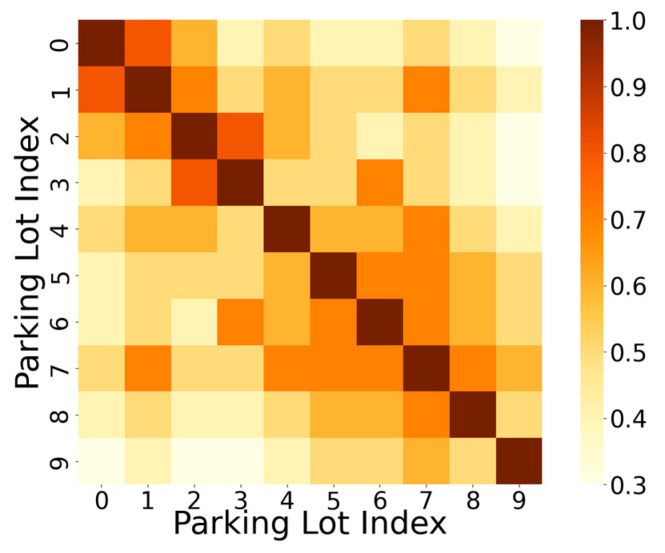
Parking lot confusion matrix.

Figure 10 displays a heatmap of parking lot traffic similarity for 10 different locations. In the comparison of prediction accuracy across different features in the model (Figure 7), we observed that removing geographic features—specifically setting the adjacency matrix generated from the latitude and longitude of each parking lot to either an all-ones matrix or an all-zeros matrix, thereby ignoring connectivity between parking lots—resulted in a decrease in prediction accuracy. This indicates that geographic features have a certain impact on parking lot traffic. To validate this observation, we selected a subset of data containing 10 identical POI parking lots in Huangpu District, Shanghai, for multiple experiments. As shown in Figure 10, each row i represents the strength of correlation between each parking lot and the i-th parking lot. For instance, it can be observed that the parking traffic of the first parking lot correlates more strongly with the seventh parking lot than with the ninth parking lot. Upon querying the specific addresses (latitude and longitude) of these three parking lots, we found that the first parking lot is adjacent to the seventh parking lot but farther away from the ninth parking lot. This demonstrates that geographic features have a certain influence on parking lot traffic, as adjacent parking lots tend to exhibit similar parking traffic. This finding not only confirms the model’s ability to achieve good predictive performance but also highlights its interpretability in practice.

**Figure 11:**
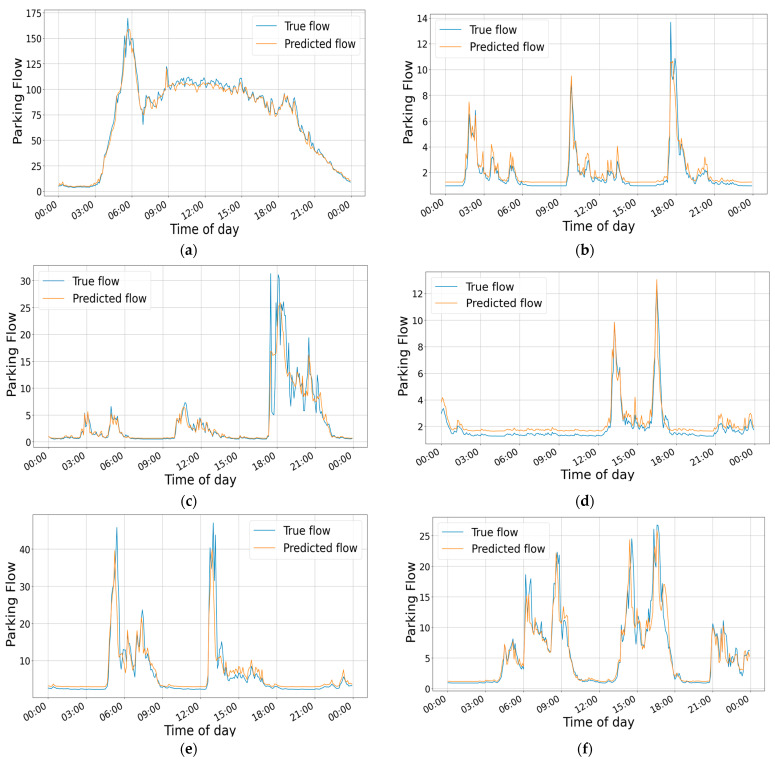
Visualization of parking lots flow prediction. (**a**) Visualization of a shopping mall parking lot flow prediction. (**b**) Visualization of a hotel parking lot flow. (**c**) Visualization of a residential area parking lot flow prediction. (**d**) Visualization of a park parking lot flow prediction. (**e**) Visualization of an office building parking lot flow prediction. (**f**) Visualization of a hospital parking lot flow prediction.

Figure 11 is a visual representation of the parking flow of parking lots in Huangpu District, Shanghai, on 15 December 2021. It depicts the typical daily parking lot traffic predictions for shopping malls, hotels, residential areas, parks, office buildings, and hospitals in the experiment. The figure clearly shows that the parking lot traffic in shopping malls is substantial, and it roughly aligns with the working hours of the staff and the arrival times of customers. After 6 p.m., there is a significant increase in parking lot traffic in residential areas, corresponding to the time when people get off work. The parking lots of office buildings exhibit obvious morning and afternoon peaks, while the parking lot traffic at hospitals is closely related to the working hours of the hospital staff. The parking lot at the hotel experiences higher traffic in the evening, whereas the parking lot at the park sees higher traffic during the daytime. These visualizations intuitively demonstrate the objective impact of external factors such as the functionality zones of the parking lot on parking lot traffic, proving the feasibility of the experimental approach and the accuracy of our MFF-STGCN model.

## 6. Conclusions

In this paper, we present a new model, MFF-STGCN, a spatio-temporal learning framework based on multifaceted spatio-temporal features (such as POI distribution and weather) for predicting parking lot traffic. We constructed a feature embedding module to model external factors influencing parking lot traffic, such as points of interest (POIs) and weather. We constructed a spatio-temporal attention mechanism layer to learn the dynamic spatio-temporal correlations of parking lot occupancy data. In this mechanism, the spatial attention mechanism models the complex spatial correlations between different parking lots to identify the degree of correlation between different locations and adjust the model’s predictions accordingly. Meanwhile, the temporal attention mechanism captures the dynamic temporal autocorrelations between different time intervals to capture the changing trends of parking lot occupancy over time. To capture the spatio-temporal features of parking lot traffic, we constructed a novel spatio-temporal convolutional module. By utilizing graph convolutions and convolutions in the temporal dimension, we aim to more accurately model and predict the spatio-temporal dependencies of parking traffic data. We conducted extensive experiments on a real-world parking lot traffic dataset, validating that our model outperforms existing baselines and achieves the best predictive performance.

We discuss the relevance of the results and the possibility of their verification in practice. Firstly, accurate prediction of parking lot traffic not only provides data support and recommendations for parking management but also offers reliable parking information to drivers, enabling them to plan optimal parking routes, enhance parking efficiency, locate parking spaces more quickly, and alleviate road congestion and associated parking-related congestion issues. Our proposed model demonstrated good performance in predicting accuracy using a dataset from parking lots in Huangpu District, Shanghai. To apply it in real-world scenarios, further validation of the model’s generalization ability across additional datasets is necessary. Secondly, real-world applications require the consideration of real-time data acquisition, noise handling, and anomaly detection. Our model includes certain data processing capabilities, yet enhancing modules for data acquisition and processing to improve data quality according to actual conditions is essential. Moreover, for large parking lots or high-traffic areas, the model needs rapid response capabilities to adapt to quick changes in parking demand. In our experiments, we adjusted model parameters to balance computational efficiency and prediction accuracy. However, achieving real-time responsiveness requires ongoing adjustments and optimizations. Thirdly, different types of parking facilities such as commercial centers, offices, and hospitals exhibit distinct parking behavior patterns and characteristics. We considered these features during model development, enabling it to possess some level of generalization and adaptability across diverse application scenarios. Finally, practical applications may necessitate simplifying the model or integrating other sensing technologies to reduce implementation costs and enhance benefits. See Section 6 for details.

Moving forward, we plan to further consider the influence of external factors such as social events. In fact, the prediction of traffic flow in parking lots is similar to the prediction of road traffic flow, and in the future, we will consider applying MFF-STGCN to the prediction of traffic flow on highways or other applications and continue research in parking lot traffic prediction and urban road traffic prediction, striving to provide convenience for residents’ travel and daily life.

## Figures and Tables

**Table 1:** Comparison of module prediction accuracy.

Model	GRU	LSTM	SSTGCN	STGCN	ASTGCN	Ours
MAE (15 min)	2.731	2.662	2.651	2.445	2.210	1.893
RMSE (15 min)	4.865	4.739	4.723	4.593	4.412	3.892
MAE (30 min)	3.076	2.983	2.874	2.798	2.362	2.16
RMSE (30 min)	5.213	5.136	5.141	4.942	4.793	4.45
MAE (45 min)	3.843	3.769	3.352	3.263	2.781	2.509
RMSE (45 min)	5.965	5.892	5.605	5.442	5.393	5.174

## Data Availability

The data presented in this study are available on request from the corresponding author. The data are not publicly available due to the presence of sensitive personal information or privacy data in the data.
